# Supplementary material for: Absorption and Biotransformation of Selenomethionine and Selenomethionine-Oxide by Wheat Seedlings (Triticum aestivum L.)
Source: Plants (Basel). 2024 Jan 27;13(3):380. doi: 10.3390/plants13030380 (PMC10857051; doi:10.3390/plants13030380)
Supplement: Supplementary file 1 [file plants-13-00380-s001.zip › plants-2783807-supplementary.pdf]

Absorption and biotransformation of selenomethionine and selenomethionine-oxide by wheat seedlings (*Triticum aestivum* L.)

Qi Wang, Siyu Huang, Qingqing Huang, Yao Yu, Huafen Li, Yanan Wan*

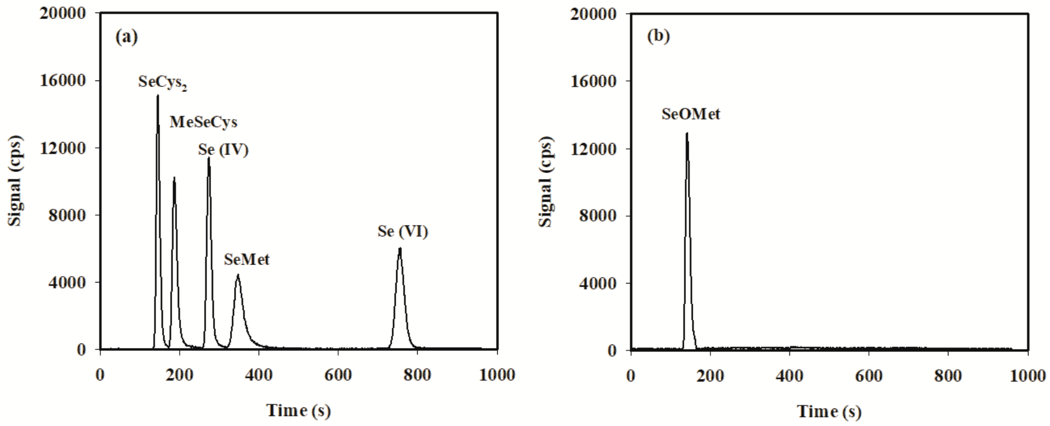

Figure S1. Analytical HPLC-ICP-MS chromatogram of six standard Se species (100 µg L^{-1}): (a) $SeCys_2$, MeSeCys, Se (IV), SeMet and Se (VI); (b) SeOMet.
